# Clinical Update on Congenital Adrenal Hyperplasia: Recommendations from a Multidisciplinary Adrenal Program

**DOI:** 10.3390/jcm12093128

**Published:** 2023-04-26

**Authors:** Thomas Uslar, Roberto Olmos, Alejandro Martínez-Aguayo, René Baudrand

**Affiliations:** 1 Program for Adrenal Disorders CETREN-UC, Pontificia Universidad Católica de Chile, Diagonal Paraguay 362, Santiago 8330077, Chile; tuslar@gmail.com (T.U.); olmos.roberto@gmail.com (R.O.); alejandro.martinez.aguayo@gmail.com (A.M.-A.); 2 Department of Endocrinology, School of Medicine, Pontificia Universidad Católica de Chile, Diagonal Paraguay 362, Santiago 8330077, Chile; 3 Division of Pediatrics, School of Medicine, Pontificia Universidad Católica de Chile, Diagonal Paraguay 362, Santiago 8330077, Chile

**Keywords:** congenital adrenal hyperplasia, non-classic CAH, hyperandrogenism, recessive genetic disorder

## Abstract

Congenital adrenal hyperplasia (CAH) is a common genetic disorder in endocrinology, especially its milder clinical presentation, often caused by a partial or total deficiency of the 21-hydroxylase enzyme located in the adrenal cortex. CAH is characterized by the overproduction of androgen, along with variable degrees of cortisol and aldosterone deficiency. The age at diagnosis can provide some information about underlying mutations, with those diagnosed at birth/early infancy more likely to have severe enzymatic defects, which may include adrenal insufficiency, sexual development disorders, short stature in adulthood, hirsutism, and a higher risk for metabolic syndrome and infertility. Non-classic CAH, a milder form of CAH, is usually manifested later in life and is a common differential diagnosis of Polycystic Ovary Syndrome and should be actively evaluated during initial studies of clinical or biochemical hyperandrogenism. The main goals of CAH treatment are hormone supplementation for severe cases, controlling adrenal androgen overproduction to minimize long-term side effects, managing fertility and genetic counseling, and optimizing patients’ quality of life.

## 1. Introduction

**Figure 1:**
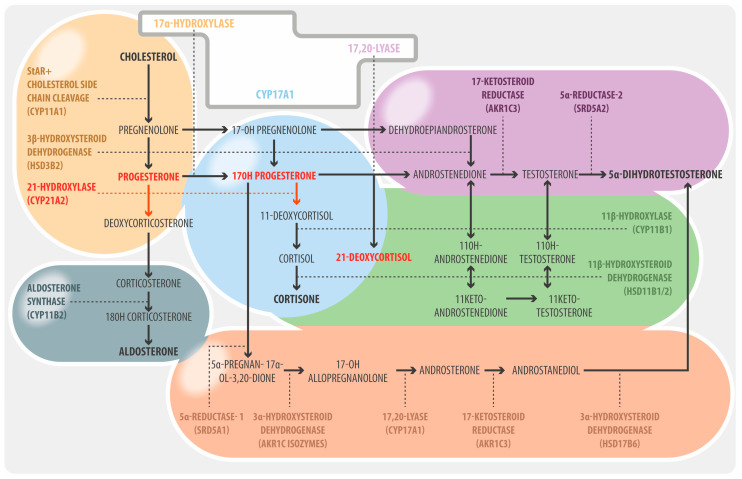
Classic adrenal steroidogenesis pathway and alternative pathways in CAH. Light orange denotes early steps of steroidogenesis common to all zones. Gray and light blue, steps that lead to aldosterone synthesis in zona glomerulosa and cortisol synthesis in zona fasciculata respectively, impaired by 21-hydroxilase deficiency. Purple, classic pathway of adrenal- and extra-adrenal androgen synthesis. Orange, “backdoor” pathway that leads to dihydrotestosterone production. Green, CYP11B1 pathway of 11-oxyandrogen production. Adapted from Claahsen-van der Grinten et al. [3].

Congenital adrenal hyperplasia (CAH) is a group of genetic disorders with a wide spectrum of clinical manifestations and is, therefore, challenging in endocrine practice. More precisely, the term CAH refers to a group of genetic disorders that affect the adrenal glands. These are autosomal recessive alterations that cause metabolic dysregulations, including impaired cortisol synthesis, due to deficient activity of enzymes located in the zona fasciculata of the adrenal cortex. In most cases (>95%), these are mutations that affect the *CYP21A2* gene, which encodes the enzyme 21-hydroxylase. Although all forms of CAH reduce cortisol production, this may occur at different degrees and various levels, affecting distinct segments of the adrenal steroid biosynthesis pathway. Reduced cortisol production in CAH patients leads to an increase in their hypothalamic CRH and pituitary ACTH levels, which stimulates adrenal cortex hyperplasia, and, in some cases, adrenocortical nodularity. In turn, given their common metabolic routes, adrenal hyperplasia leads to an increase in the synthesis of other steroid hormones. Hence, despite the lack of cortisol biosynthesis, CAH can paradoxically cause adrenal hyperfunction affecting other steroid pathways [1,2] (Figure 1).

## 2. Epidemiology

Although its incidence depends on ethnic (inbred) and geographic factors, classic CAH (caused by D21OH) is a rare disease with a prevalence that ranges from 1 in 10,000 to 1 in 16,000 cases [2]. In contrast, non-classic CAH (NCCAH; also called late-onset CAH) has a much higher frequency (up to 1/500); NCCAH is also common within ethnic groups with high consanguinity, such as “Ashkenazi” Jews. Worldwide, CAH (including mild cases) is considered the most common autosomal recessive disease, surpassing cystic fibrosis and phenylketonuria [4].

## 3. Pathophysiology and Classification of Congenital Adrenal Hyperplasia Due to 21-Hydrolase Deficiency

**Figure 2:**
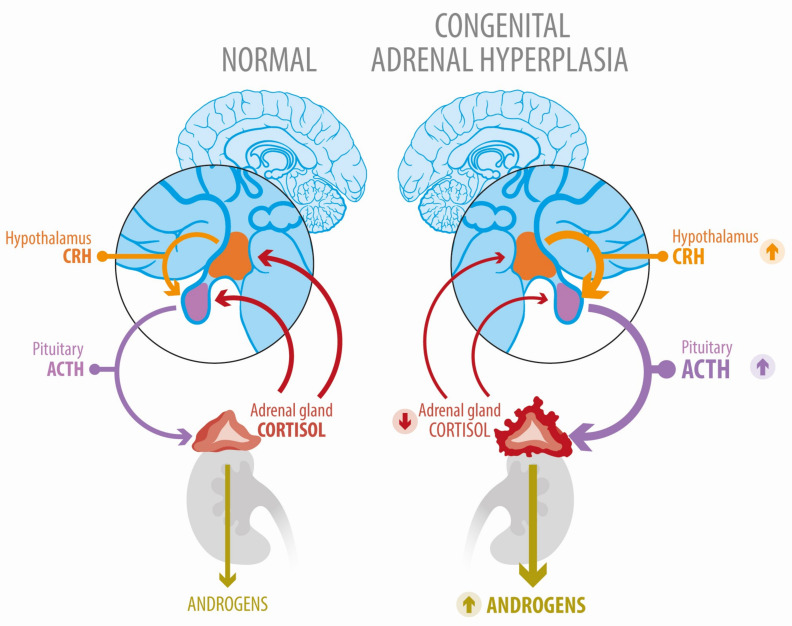
Deregulated hypothalamic–pituitary–adrenal axis function in congenital adrenal hyperplasia due to 21-hydroxylase deficiency.

As explained, alterations in the CYP21A2 gene translate into enzymatic deficiency of the 21-hydroxylase (21OHD) activity causing a decrease in cortisol biosynthesis. However, in certain severe cases, CYP21A2 mutations can also affect aldosterone production. Then, the lack of cortisol feedback results in increased ACTH, which promotes the accumulation of 17-hydroxyprogesterone (17OHP) and other steroids that serve as substrates for androgen excess (Figure 1). Allelic CYP21A2 variants are associated with a continuum spectrum of enzyme phenotypes. As pointed out, CAH is a recessive genetic disorder. Therefore, affected subjects can be either homozygous (same mutation affects both alleles) or compound heterozygous (two different mutations affecting each allele). Importantly, the onset and severity of the clinical manifestations of the disease will ultimately depend on the allele affected by the “mildest” mutation [5]. Clinical CAH phenotypes are characterized by decreased cortisol synthesis and increased androgen secretion and depend on both the age at presentation and the severity of the CYP21A2 mutation. Hence, CAH cases can be divided into three categories [2]: (a) Salt-wasting (SW) represents 65–75% of the classic CAH cases. These are manifested in infancy and are characterized by a marked cortisol and aldosterone deficiency along with hyperandrogenism. Residual enzymatic activity of SW-CAH is typically < 1%. (b) Simple virilizing CAH comprises 25–35% of the classic CAH. Unlike SW-CAH, this form manifests later in life and is characterized by a severe cortisol deficit, but unaltered aldosterone. Residual enzymatic activity of this form of CAH is 1–2%; and (c) Non-classic CAH is the most frequently seen in the clinic. It usually manifests during puberty and is characterized by hyperandrogenism. Residual enzymatic activity ranges from 20% to 50% [6] (Figure 2) and normal cortisol production is maintained by excess ACTH.

**Figure 3:**
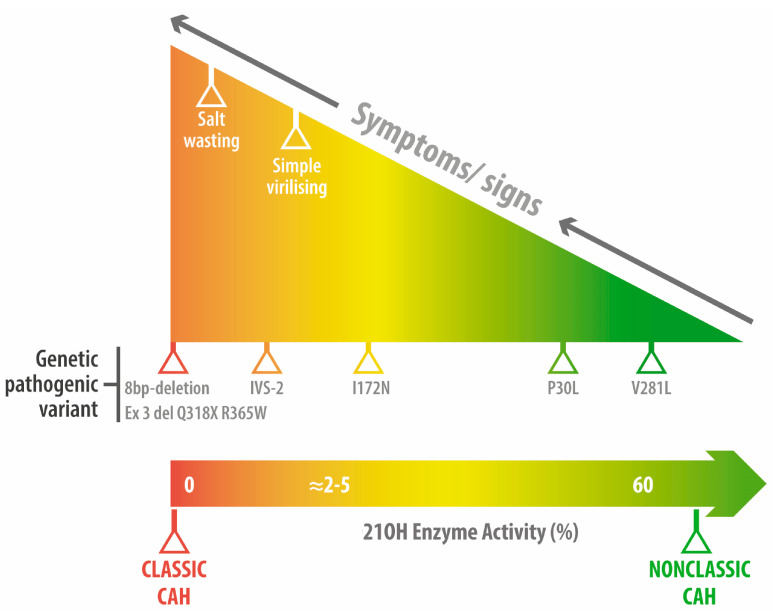
Most frequent pathogenic variants in adrenal hyperplasia due to 21-hydroxylase deficiency.

As noted, CAH subtypes are determined by the severity of underlying mutation(s). For example, a heterozygous individual that harbors a “severe” and a “mild” mutation will fall into the NCCAH subtype. Therefore, an individual that harbors a “severe” mutation but also carries a wild-type allele will not manifest the disease [7] (Figure 3).

## 4. Clinical Features

### 4.1. SW Classic CAH

Severe aldosterone and cortisol deficiency in this condition can lead to a salt-wasting crisis in newborns. However, this is unlikely to occur within 5 days of birth, which is usually when newborns are discharged from the hospital. Therefore, SW-CAH requires high diagnostic suspicion, particularly among males due to the lack of genital ambiguity. Affected individuals typically present with adrenal insufficiency associated with hypovolemic shock, hyponatremia, hyperkalemia, metabolic acidosis, and sometimes hypoglycemia. In 46, XX individuals, this condition is accompanied by various degrees of virilization due to exposure to excess androgens during intrauterine development. Although the uterus, fallopian tubes, and ovaries are formed normally, XX DSD may involve clitoral hypertrophy, labial fusion, and urogenital sinus defects. The Prader classification ranges from 1 to 5, where grade 1 displays mild clitoral hypertrophy and grade 5 is characterized by complete masculinization. Excess adrenal androgens do not affect 46, XY sexual differentiation.

### 4.2. Simple Virilizing CAH

This condition is usually detected before puberty and evidenced by clinical signs, such as hyperandrogenism, premature puberty/adrenarche, apocrine odor, precocious puberty, clitoromegaly, rapid growth, and accelerated skeletal maturation (with a compromise of final height). Most patients display subclinical cortisol deficiency but preserve their mineralocorticoid function [2,8].

### 4.3. Non-Classical CAH

**Figure 4:**
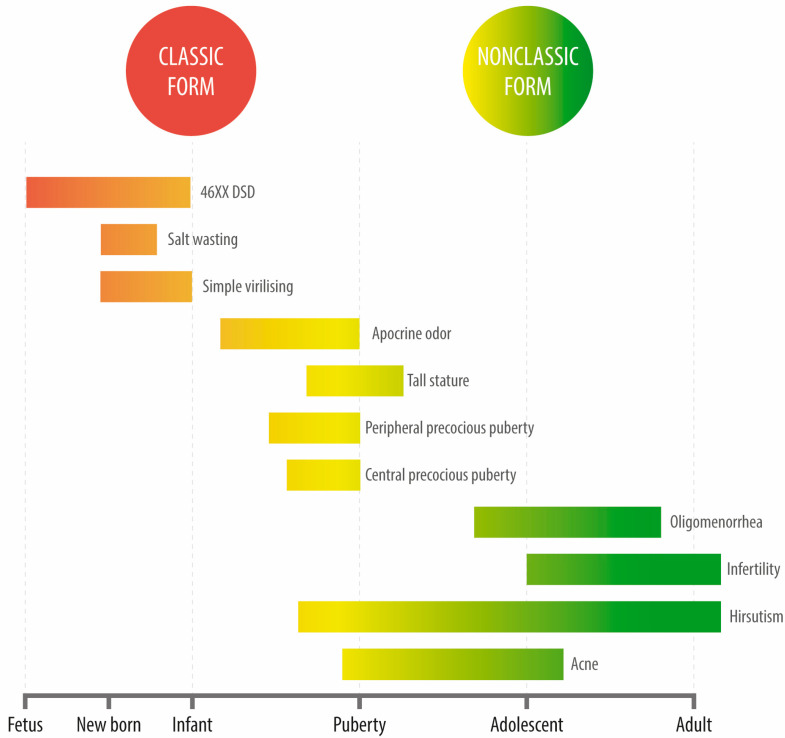
Clinical manifestations of congenital adrenal hyperplasia due to 21-hydroxylase deficiency at different ages.

Unlike classic CAH, cortisol and aldosterone levels in these patients remain unaffected. The most frequent reason for consultation among these patients is late-onset hyperandrogenism, generally manifested either in the peri-pubertal stage or during adulthood. This condition is severely underdiagnosed among males given the evident difficulties in assessing hyperandrogenism. During adolescence, the most frequent reasons for consultation are acne, hirsutism, or oligomenorrhea (a condition that is clinically indistinguishable from polycystic ovary syndrome (PCOS)). Affected individuals may also display polycystic ovarian morphology. Oftentimes, NCCAH diagnosis occurs later in life, even into adulthood, in the context of infertility studies or after recurrent miscarriages (Figure 4).

## 5. Initial Studies

### 5.1. Steroid Analyses

The initial diagnosis of CAH is commonly based on plasma 17OHP levels. Enzymatic deficiency in these individuals leads to the accumulation of this steroid hormone in direct proportion to the severity of the defect. Early detection and treatment of 21OHD-related CAH can prevent serious morbidity and mortality; therefore, all newborn screening programs should incorporate these tests [6]. In locations where universal screening is unavailable, 17OHP should be measured in newborns/infants that display clinical signs/symptoms and in women with suspicion of polycystic ovary syndrome, hirsutism, and/or infertility.

Measurement techniques for 17OHP include immunoassays and liquid chromatography with tandem mass spectrometry (LC-MS/MS). Each of these may determine different cut-off values that must be adjusted by age and sex. International guidelines recommend LC-MS/MS given its specificity and accuracy [6,9,10]. Due to the circadian variation in adrenal hormones, this test requires fasting and must be taken before 9:00 AM. In addition, since the corpus luteum produces 17OHP, this test should be taken during the early follicular phase in women with NCCAH. This is not the case for users of oral contraceptives (OCP), where 17OHP can be measured at any time due to the absence of the luteal phase. 

**Figure 5:**
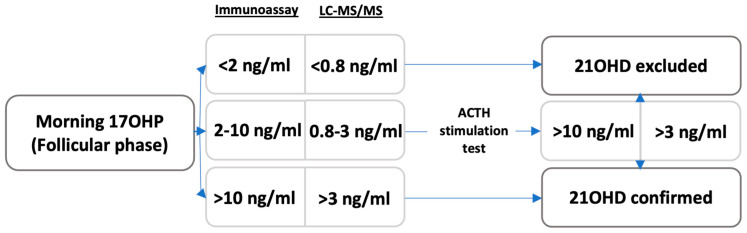
Diagnosis algorithm of 21OHD. Reference standards for hormonal diagnosis were derived from Refs. [6,10].

In general, >2 ng/mL plasma 17OHP by immunoassay is considered suspicious, while >7–10 ng/mL is a consensus for CAH diagnosis. For intermediate cases, the current recommendation is to perform a stimulation test with ACTH (intramuscular or i.v., 250 ug) to confirm the diagnosis. For LC-MS/MS measurements, >0.8 ng/mL of plasma 17OHP is considered elevated (suspicious) while >3 ng/mL 17OHP levels following ACTH stimulation is enough to confirm the diagnosis [10] (Figure 5).

In addition to its utility as a confirmatory test for CAH diagnosis, ACTH stimulation allows the measurement of the cortisol response in NCCAH patients. In many cases, this subgroup requires corticosteroid supplementation under stress. In the case of nondiagnostic stimulated 17OHP values, especially in those receiving glucocorticoid therapy, and the unavailability of an ACTH stimulation test, genotype analysis should be used to confirm CAH. We expect that, in a future with lower costs and better availability, genetic testing will help to confirm a diagnosis in a complementary way to the ACTH test, especially in cases with one severe mutation allele carrier that have indeterminate 17OHP values but do not have the disease and need genetic counseling.

Lastly, whenever there is suspicion of classic CAH, other adrenal steroids, such as cortisol, aldosterone, androstenedione, and DHEAS, should also be assessed. This includes the mineralocorticoid axis, which should be evaluated by assessing plasma renin and plasma electrolytes.

### 5.2. Genetic Aspects of Diagnosis

Genetic studies are highly recommended for all cases where biochemical analyses suggest CAH. These are also essential to confirm the diagnosis in difficult cases, as a critical determinant of prognosis, and to deliver accurate genetic counseling [11,12]. The gene that encodes the enzyme 21-hydroxylase, known as *CYP21A2*, is located on the short arm of chromosome 6 (6p21.3). This is a particularly complex chromosomal region due to the presence of highly homologous pseudogenes. As a result, almost 75% of 21-hydroxylase pathogenic variants are caused by micro-conversions between *CYP21A1P* and *CYP21A2,* while the remaining 20–25% are the result of unequal crossing-overs that occur during meiosis, and only 1–2% correspond to spontaneous mutations not inherited from progenitors [7]. 

Although there are >200 pathogenic variants of 21-hydroxylase reported to date, a small subset of 10 of these variants account for almost 90% of CAH cases (Figure 2). As mentioned above, two-thirds of NCCAH cases have a genotype that includes one severely mutated allele accompanied by a mildly mutated allele (compound heterozygotes). Given the potential risk of having a child with classic CAH, these patients require genetic counseling. This may occur if one parent is a carrier of a severe allele, which is present in around 1 out of 60 asymptomatic individuals. Genetic counseling can help these patients to make informed decisions regarding their family planning and at the same time reduces the risk of passing this condition on to their offspring.

In classic CAH, there is a strong genotype-to-phenotype correlation. However, in NCCAH, this correlation is not as strong. Consequently, NCCAH management should be primarily based on clinical and hormonal data [3]. Based on their enzymatic activity, *CYP21A2* mutations are classified into three groups. Group A includes severe “nonsense” mutations and deletions that result in a protein with no enzymatic capacity. These mutations/alterations are associated with the earliest and most severe forms of the condition (SW-CAH). Group B comprises less severe mutations that retain 1 to 10% of the residual enzymatic activity and typically manifest as SV-CAH. Finally, Group C contains mutations that conserve 20 to 60% of the enzymatic capacity and are typically found in NCCAH (Figure 2) [3].

## 6. Treatment of CAH

### 6.1. Classic CAH

The treatment of classical CAH involves achieving a balance of three key clinical goals: to promote the physiological replacement of adrenal insufficiency, to reduce the exposure to age- and sex-appropriate levels of adrenal androgens, and to avoid iatrogenic hypercortisolism and its potentially associated comorbidities [13].

Glucocorticoid replacement clinical recommendations: Hydrocortisone is typically the preferred choice and should be administered two or three times per day, with higher morning and lower evening doses. Children usually require 10–15 mg/m^2^ per day every 8 h, while adults may require 15–25 mg per day with a last dose administered at least 6 h before bedtime to maintain the circadian rhythm. 

In newborn patients suspected of having the salt-wasting (SW) form of CAH due to high levels of 17OHP in the screening sample, urgent clinical advice is necessary for appropriate treatment. The initial dose of hydrocortisone given to neonates and infants with a newly detected SW form should be adjusted based on their clinical situation. If the newborn has elevated potassium and decreased sodium levels, prompt treatment should begin, including intravenous glucose infusion containing sodium and intravenous hydrocortisone. The initial hydrocortisone bolus dose suggested is 5 mg/kg, followed by 25 mg per 24 h as a continuous infusion or divided into three or four doses. As children grow older, the recommended hydrocortisone dose for CAH typically increases. Children may require a daily dose of 10 mg/m^2^ every 8 h. For adults with CAH, the recommended dose may range from 15 to 25 mg per day, with the last dose administered at least 6 h before bedtime to maintain the circadian rhythm [14,15].

In cases where ACTH remains excessively elevated despite treatment, a low dose of nocturnal prednisone (1–2 mg) can be prescribed, especially after puberty [13]. In cases where a patient is struggling with adherence to evening hydrocortisone or experiencing hyperpigmentation, the alternative is a single morning dose (5–7.5 mg/d) of long half-life glucocorticoids, such as prednisone. In contrast, dexamethasone (0.25–0.5 mg/d dose) is not recommended given the difficulty of titration and the risk of an iatrogenic Cushing’s syndrome [6]. It is noteworthy that monotherapy with nocturnal corticosteroids is not appropriate in these cases as they can lead to long-term sleep and/or mood disorders. Of note, long-acting glucocorticoids could lead to detrimental effects on the growth of children with severe forms of CAH [16,17]. In children, the growth velocity, weight, blood pressure, bone age, signs of hyperandrogenism, and the presence of adrenal remnants in the testes should be closely monitored by ultrasound [6,13].

Mineralocorticoid replacement clinical recommendations: Fludrocortisone is often the drug of choice. Typically, it is administered in a single morning dose that ranges from 0.05 to 0.2 mg/day (with salt-free intake). In addition, most formulations do not require strict refrigeration. However, it is important to note that neonates and young infants may require higher doses of fludrocortisone and may also need sodium chloride supplementation to maintain a proper sodium balance. The fludrocortisone dose should be titrated to reach a plasma renin activity within the normal/high range that also ensures normokalemia [3].

In rare cases where medical management cannot effectively control hyperandrogenism, or in cases where its control would require producing iatrogenic hypercortisolism, bilateral adrenalectomy may be considered. This option is common for adenomas and large bilateral myelolipomas secondary to ACTH stimulation [18].

#### 6.1.1. Treatment under Medical-Surgical Stress Conditions

Classic CAH patients are at a high risk of suffering adrenal insufficiency crises manifested as hypoglycemia, seizures, hyponatremia, hyperkalemia, dehydration, and/or refractory shock. To prevent these potentially life-threatening events, all the affected patients should be informed about these risks and carry identification in the form of a bracelet or a badge. Patients should also maintain emergency corticosteroids at hand in case they are unable to take their regular oral medication due to complications, such as vomiting or severe diarrhea. Notably, certain situations, such as fever (>38.5 °C), tooth extraction, simple outpatient surgery, or endoscopic procedures can increase the risk of adrenal insufficiency crises. For these cases, the recommendation is to double the usual corticosteroid dose to prevent these events [19].

#### 6.1.2. New Drugs to Improve the Treatment of CAH Patients

Recent research has sought to replicate the circadian rhythm of glucocorticoid supplementation to reduce exposure to these compounds. Modified or delayed-release hydrocortisone formulations and continuous subcutaneous delivery of hydrocortisone may be useful for patients with a rapid cortisol metabolism or impaired gut absorption. However, the long-term clinical utility beyond improved biochemical parameters is still uncertain [3]. In pre-pubertal CAH patients, medications that reduce androgen production and/or its action, such as testolactone, flutamide, and abiraterone acetate, may be useful for androgen suppression until the anticipated age of puberty. However, further studies are necessary to confirm their effectiveness and safety. Lastly, CRH antagonists have recently emerged as promissory treatments in patients with poor disease control CAH [20].

#### 6.1.3. Restoring Functional Anatomy in 46, XX Subjects Virilized by Classic CAH

For 46, XX virilized individuals with classic CAH, surgery is not a medical emergency unless there are urogenital sinus complications. These cases should seek treatment exclusively at referral centers with pre-established protocols and a multidisciplinary team of professionals specialized in DSD management [6]. 

#### 6.1.4. Preventing Prenatal Virilization in At-Risk Pregnancies

For >40 years, dexamethasone has been proposed as a treatment to prevent the virilization of a female fetus that inherits two severely mutated CYP21A2 alleles from her mother with classic CAH during pregnancy. Although the main goal of dexamethasone is to suppress fetal ACTH production, its use can result in pharmacological hypercortisolism, affecting both the mother and the fetus since dexamethasone is not inactivated in the placenta. Studies recommend a preconception dose of 20 ug/kg and up to 1.5 mg which is 2–6 times the maternal physiological requirement and almost 60 times that of the fetus. Although this intervention is successful in 85% of cases, effectively reducing the Prader score by about 40% [21], it is important to weigh up its risks and benefits. Some of these include teratogenicity in animals (class C drug) and maternal Cushing’s syndrome [22]. Importantly, it should be noted that seven out of eight fetuses will be treated unnecessarily, at least until the biological sex of the individual is known. Furthermore, given the effectiveness of genital surgery, current international guidelines do not recommend routine prenatal dexamethasone treatments. This intervention should be considered rather as an experimental procedure performed at tertiary centers after obtaining informed consent signed by both parents and the approval of an ethics committee [6]. The current recommendation to avoid the unnecessary treatment of male fetuses is to perform early measurements of the fetal Y chromosome in maternal blood, starting at 6–8 weeks of gestation.

### 6.2. NCCAH

By definition, NCCAH does not involve adrenal insufficiency. Therefore, in most cases, cortisol replacement is neither necessary nor recommended, especially in adult individuals. Despite this, the current guidelines from the Endocrine Society recommend cortisol replacement in two specific cases: (1) in adult women with hyperandrogenism and fertility disorders and (2) in pediatric patients with rapidly progressive precocious puberty associated with advanced bone age. Nonetheless, in patients with central precocious puberty, it is possible to halt puberty at the hypothalamic level using GnRH agonists, avoiding the use of corticosteroids and their potential adverse effects [6]. 

As mentioned earlier, NCCAH often affects adolescent or adult women with acne and hirsutism. As with PCOS, the typical first-line treatment is a combination of oral contraceptives. In patients that display a poor response to treatment, antiandrogens, such as spironolactone (initial dose 50–100 mg/day) or low doses of flutamide (62.5–125 mg/day), may be added to the treatment regimen [6].

## 7. Special Clinical Situations

### 7.1. Infertility and Pregnancy

#### 7.1.1. Classic CAH

Up to 90% of women with untreated CAH experience infertility; this is linked to the severity of the *CYP21A2* allelic variant. High levels of adrenal precursors may be aromatized to estrogens, suppressing the hypothalamic–pituitary–gonadal axis. The optimization of the corticosteroid dosage can improve ovulation rates by reducing circulating progesterone levels during the follicular phase. Other aspects, such as anatomic distortion, delayed gender assignment, and altered psychosocial development should be addressed to ensure the overall well-being of patients [23].

#### 7.1.2. NCCAH

Infertility and a higher rate of miscarriage are common among women with NCCAH. These complications can be improved using hydrocortisone periconceptionally and during pregnancy, achieving conception rates close to 90%. As increased adrenal production of progesterone in the follicular phase is one of the causes of infertility in NCCAH, treatment should aim to maintain progesterone levels below 0.6 ng/mL [24].

### 7.2. Genetic Counseling

Preconception counseling is crucial for patients at risk of having a child with classic CAH. As mentioned previously, this condition occurs when both parents have at least one severe allelic variant. In the general population, the estimated prevalence of severe allelic variants is 1 in 60 (2%). To provide adequate counseling, both parents should undergo genetic and biochemical testing. If the tests show that the partner does not carry a severe allelic variant, or if the woman harbors a milder variant, pregnancy can be attempted without a risk of classic CAH for the offspring. However, the risk of NCCAH still persists [9].

### 7.3. Testicular Adrenal Rest Tumors (TARTs)

Classic CAH may impair male reproductive function due to the presence of testicular steroid cell masses. These masses locate near the testicular mediastinum and respond to ACTH, which can have a mechanical and paracrine effect on spermatogenesis (21). The prevalence of TARTs can be as high as 20% among children and between 50 and 80% in adults with classic CAH. An expert recommendation for boys with classic CAH is to perform testicular ultrasound starting at the age of 8 years and conduct follow-up scans every 2 years. The treatment for TARTs consists of optimized pharmacological management. Surgery is only indicated for cases of severe pain complaints, as it has been proven ineffective in restoring fertility by retrospective cohorts [25].

### 7.4. Behavioral Considerations in Patients with Classical CAH

Classic CAH may be associated with a wide spectrum of features ranging from generic factors, such as psychiatric symptomatology observed in other chronic medical conditions, to CAH-specific factors, such as behavior and interests more typical of males than females (also described as “tomboy” behavior) [26]. The strength of binary gender identity in affected females may be reduced. However, this does not necessarily imply that this condition changes their gender identity or sexual orientation. While prenatal androgen exposure may contribute to the occurrence of these outcomes, its influence is much smaller than its effect on gender-role behavior [3]. 

## 8. Follow-Up

Surveillance in CAH patients should focus on several areas that include (a) clinical and biochemical monitoring of hormonal replacement therapy, (b) the correction of hyperandrogenism, (c) the monitoring of height and anthropometry in pediatric patients, and (d) the assessment of the potential side effects of therapy. For patients with classic CAH or women with NCCAH who experience fertility issues, we recommend treatments delivered by a multidisciplinary team with expertise in this condition, including endocrinologists, gynecologists, and geneticists [23]. Laboratory tests should always be performed during therapy. Androstenedione, and occasionally 17OHP, should be targeted to the upper range of the normal reference or a slightly elevated value, ideally measured by LC-MS/MS. In women with CAH seeking pregnancy, or in men with TARTs, lower androstenedione levels are recommended. It is important to note that normalizing ACTH is not a therapeutic goal, as this would indicate overtreatment with glucocorticoids. Regarding follow-up recommendations for adults with classic CAH, annual measurements of blood pressure and monitoring of the body mass index (BMI) and plasma renin levels are advised, with a therapeutic target for renin at the upper normal limit [6].

Finally, ensuring that patients receive ongoing education about their disease is critical for long-term care and follow-up. This includes education about the use of wearing an identification bracelet or a tag and providing guidance on dose adjustment in stressful situations, as well as periodical assessments of patients’ quality of life [3,6].

## 9. Future Perspectives

CAH is a complex and diverse condition that demands expert-level comprehensive care. Due to its prevalence and severity, we advocate for mandatory newborn screenings for CAH worldwide. Although non-specialists may manage NCCAH, the classic forms require evaluations at specialized multidisciplinary centers. The long-term management of the disease should focus on risk assessments for adverse outcomes because of hormonal excess, minimizing glucocorticoid overexposure in patients. In this regard, hormone supplementation has made significant progress in recent decades, and newer hydrocortisone formulations that replicate the normal circadian rhythm along with other novel modalities are currently under development. Looking forward, the goal is to achieve more patient-centered psychobiological treatment modalities that address both socioeconomic and cultural issues.

**Methods of Bibliographic Research:** The authors, who are experts in adrenal disorders, searched for studies published up to October 2022. Multiple databases were searched, including MEDLINE, Cochrane, and EMBASE. The terms of the MEDLINE search were (classic OR classical) AND (non classic OR non-classic OR late-onset OR late onset) AND (adrenal hyperplasia OR 21-hydroxylase OR 21α-hydroxylase OR CYP21 OR CYP21A2). Certain studies were excluded from the review process for various reasons, such as the data being unrelated to the research focus, insufficient data for epidemiological analysis, or data already reported in previous publications. All the data sources were analyzed while recognizing positive publication bias.
